# Heterologous Overexpression of Arabidopsis *cel1* Enhances Grain Yield, Biomass and Early Maturity in *Setaria viridis*

**DOI:** 10.3389/fpls.2020.515078

**Published:** 2020-11-10

**Authors:** Bala P. Venkata, Robert Polzin, Rebecca Wilkes, Armahni Fearn, Dylan Blumenthal, Sara Rohrbough, Nigel J. Taylor

**Affiliations:** Donald Danforth Plant Science Center, St. Louis, MO, United States

**Keywords:** *Setaria viridis*, enhanced grain yield, Arabidopsis *cel1*, shoot biomass, accelerated maturity, climate smart agriculture

## Abstract

Heterologous overexpression of Arabidopsis cellulase 1 (*Atcel1*) results in enhanced yield, early maturity, and increased biomass in dicotyledonous species like poplar and eucalyptus but has not been demonstrated in monocots. We produced transgenic *Setaria viridis* accession A10.1 plants overexpressing a monocotyledonous codon optimized (*MCO) Atcel1*. Agronomic characterization of the transgenic events showed that heterologous overexpression of *MCOAtcel1* caused enhanced grain yield, shoot biomass, and accelerated maturation rate in the model grass species *S. viridis* under growth chamber conditions. The agronomic trait differences observed were consistent with previous reports in dicots but are here described in a monocot species and associated with increased seed yield. Overexpression of *Atcel1* in *S. viridis* was shown to increase the number of panicles and seeds by 24–30%, enhance overall grain yield by up to 26%, and lead to a shoot dry biomass increase of 16–19%. Overexpression also reduced time to plant maturation and senescence by 12.5%. Our findings in *S. viridis* suggest that manipulation of *Atcel1* has potential for developing early-maturing and higher-yielding monocotyledonous biomass crops suitable for climate-smart agriculture.

## Introduction

By 2050 the human population is estimated to reach 9.15 billion, with food production having to increase by 60% to meet demand (Long et al., 2015). These goals must be met in the face of challenges presented by a changing climate. Achieving global food security therefore requires the development of staple crop cultivars with superior yield potential and stress adaptation. Wheat, rice, maize, pearl millet, and sorghum provide approximately 44% of the calories consumed per capita throughout the world (Reynolds et al., 2016). Securing higher yields through the enhancement of components such as grain yield, grain number, panicle number, and panicle length in these primary cereal crops is critical (Beche et al., 2014). Early maturity is a desirable trait as it reduces potential for exposure to post-anthesis biotic and abiotic stresses, thereby providing farmers with greater flexibility when faced with uncertain growing conditions (Vadez et al., 2012).

Heterologous overexpression of plant Endo-1, 4-β- glucanase (EGase) had been reported to result in enhanced yield, early maturity, and increased biomass in poplar and eucalyptus (Shani et al., 2004; Ledford, 2014). Plant EGases are included in the glycosyl hydrolase family 9 (GH9) and divided into three distinct classes (Urbanowicz et al., 2007). At least 25 EGases have been identified in *Arabidopsis thaliana*, categorized into membrane-anchored (class A), cell wall-targeted (class B), and carbohydrate binding (class C) sub-families (Urbanowicz et al., 2007). Arabidopsis cellulase 1 (*Atcel1*) belongs to GH9B subfamily and is thought to be involved in cell wall loosening that drives anisotropic cell enlargement; a fundamental process required for plant growth (Lipchinsky, 2013). Heterologous expression of *Atcel1* in poplar and eucalyptus resulted in increased height, leaf area, biomass, dry weight, and accelerated maturation (Shani et al., 2004; Ledford, 2014). Similarly, expression of poplar cellulase *PaPopCel1* promoted enhanced biomass and early maturity in Arabidopsis and sengon (*Paraserianthes falcataria*) (Park et al., 2003; Hartati et al., 2008). No such reports exist for overexpression of *cel1* in the cereals. We performed studies in the monocot model *Setaria viridis* to investigate if heterologous overexpression of *Atcel1* can promote similar enhancements in yield, biomass, and maturation rate in a monocotyledon.

## Materials and Methods

### Identification of *Atcel1* (AT1G70710.1) Homologs in *Setaria italica*

Bioinformatics analysis^1^ of the *S. italica* genome was conducted using AT1G70710.1 amino acid sequence as a query to identify *Atcel1* homologs. Additional phylogenetic analysis was performed using phylogeny.fr (Dereeper et al., 2008) to illustrate the parallel relationship between *Atcel1* and its closest *Setaria* homolog.

### Gene Cloning and Construction of Transformation Vectors

*Sicel1* cDNA (GenBank ID Si016976m.g), the closest monocot homolog of AT1G70710.1 (*Atcel1*) was cloned from panicle tissue of 6-week old *S. italica*, Yugu-1 accession. Total RNA was isolated using TRIzol reagent (Ambion, Austin, United States) following manufacturer’s protocol and quantified using the Nanodrop2000 (Thermo Fisher Scientific, Austin, United States). One microgram of RNA was treated with DNase I (Ambion, Austin, United States) and reverse transcribed using the SuperScript III first strand cDNA synthesis kit (Invitrogen, Waltham, United States). Full-length cDNA (1509 bp) was PCR amplified by high fidelity TaKaRa (Clontech, Mountain View, United States) Taq polymerase, employing a two-step TaKaRa protocol (98°C for 3 min, 30 cycles of 98°C for 10 s and 68°C for 2 min, and 72°C for 10 min) using the specific primers *Sicel*1F (5′ATG CCG GCG GCG GTG CGG AG-3′) and *Sicel*1R (5′TCA GTC GCC GCC CGA CTC GG-3′). The 1509 bp full-length transcript was verified by *Kpn*1-HF restriction digestion (New England BioLabs, Ipswich, United States) and cloned into the *pCR8/GW* vector (Invitrogen, Waltham, United States), following the Gateway^®^ cloning protocol. After sequence confirmation (Eurofins Scientific, St. Louis, United States) the *pCR8/GW/Sicel1* clone was used as a template to recombine the gene insert into the GW^®^ compatible *pANIC10A* overexpression vector (Mann et al., 2012), using LR clonase enzyme (Invitrogen, Waltham, United States). Similarly, a 1500 bp *MCOAtcel1* coding sequence was designed and obtained from GenScript Inc., and transferred to the *pANIC10A/MCOAtcel1* vector employing the GW^®^ cloning strategy described above. The successful cloning of *Sicel1* and *MCOAtcel1* into *pANIC10A* vector was confirmed by sequencing (Eurofins Scientific, St. Louis, United States).

### Production of Transgenic *S. viridis* Plants

The coding sequences of *Sicel1* (1509 bp) and monocot codon optimized (*MCO) Atcel1* (1482 bp) were fused to the constitutive maize ubiquitin (ZmUBI) promoter and cloned into binary vector pANIC10A (Mann et al., 2012). Sequence confirmed *pANIC10A/MCOAtcel1* and *pANIC10A/Sicel1* constructs plus the empty vector control (EVC) *pANIC10A/EVC* were transformed into *Agrobacterium tumefaciens* strain AGL1 and used to generate transgenic plants of *S. viridis*, accession A10.1 (Van Eck and Swartwood, 2015). Hygromycin resistant T_0_ plantlets were transferred to soil when at least 4 cm tall with well-developed roots system (Van Eck and Swartwood, 2015). Plantlets were washed in water to disassociate growth media from the roots, planted in 3-inch pots filled with Metro-Mix 360-soil mix (Hummert International, St. Louis, United States) and watered until saturated. A plastic dome was placed over the plantlets for 3 days after which plants were grown in a Conviron (Pembina, United States) growth chamber at 12 h day/12 h night photoperiod, 31°C day and 22°C night temperature with a relative humidity of 50–60%. Plants were watered daily and fertilized twice a week with 100 ppm of N. T_0_ plantlets were grown to maturity and selfed to obtain T_1_ seeds for further characterization. T_1_ generation *S. viridis* seeds were subjected to liquid smoke treatment to break dormancy (Sebastian et al., 2014) and propagated along with wild-type A10.1 seeds under the conditions described above.

### Analysis of Transgenic Plants

T_1_ plants were genotyped 10–15 days after germination. Three hundred milligrams of leaf tissue obtained from the third fully expanded leaf was collected, placed in a 1.5 ml screw top tube and stored at −80°C. Frozen leaf tissue was crushed using TissueLyser II (QIAGEN, Germantown, United States) for 90 s at 30 Hz. One hundred milligrams of crushed leaf tissue was used for DNA isolation following the CTAB method (Fulton et al., 1995), and the remaining tissue powder saved for RNA extraction. Isolated DNA was re-suspended in 50 μl sterile Milli-Q water, quantified with a NanoDrop 2000 (Thermo Fisher Scientific, Austin, United States) and used for PCR analysis and Southern blot hybridization. RNA isolation from 200 mg of crushed leaf tissue was performed using the RNeasy Plant Mini Kit (QIAGEN, Germantown, United States), isolated RNA was quantified using the Nanodrop2000, and then treated with DNase I (Thermo Fisher Scientific, Austin, United States) following manufacturer’s protocol. One microgram of DNase I treated RNA was used to synthesize cDNA employing the SuperScript III first strand cDNA synthesis kit (Invitrogen, Waltham, United States) and stored at −20°C.

*Setaria viridis* plant lines transformed with *pANIC10A/MCOAtcel1, pANIC10A/Sicel1*, and *pANIC10A/EVC* were genotyped for presence of the hygromycin phosphotransferase (*hpt)* marker gene using primers *hpt*F (5′-GAA CTC ACC GCG ACG TCT GTC GAG-3′) and *hpt*R (5′-AAT GAC CGC TGT TAT GCG GCC AT-3′). Additionally, for plants transformed with *pANIC10A/MCOAtcel1*, presence of the *MCOAtcel1* transgene was determined using primers *MCOAtcel*1F (5′-GAA GTG GGG CAC TGA CTA CC-3′) and *MCOAtcel1*R (5′- GTA TCC ATG TCC TCA GGC CG-3′). PCR amplification was performed in a 15 μl volume containing 1 μl of 1:5 diluted cDNA template, 1× Choice Taq Blue Master mix (Denville Scientific Incorporated, Holliston, United States), nuclease-free water, and 0.35 μM each of forward and reverse primers. The PCR conditions were: initial denaturation (94°C for 5 min), 35 cycles of amplification (denaturation 94°C for 45 s, annealing 55°C for 30 s, elongation 72°C for 30 s), and final elongation (72°C for 10 min). PCR products were resolved on 1.5% ethidium bromide stained agarose gels.

### Southern Blot Analysis

Non-radioactive labeled DIG (Digoxigenin) Southern blot hybridization was used to determine T-DNA insert copy number in *S. viridis* plant lines (Brutnell et al., 2010). Ten micrograms of pooled DNA from *hpt* PCR positive T_1_ families/event for each construct was digested with *Mfe*1 restriction enzyme at 37°C for 4 h. Digested DNA was resolved on 1% agarose gel and transferred to a positively charged nitrocellulose membrane overnight, UV-crosslinked, and prepared for hybridization (pre-hybridization) using the DIG Easy Hyb (Roche, Branford, United States) solution. A probe was prepared complimentary to the *hpt* gene with DIG-labeled dNTPs and *pANIC10A* plasmid DNA as template, using forward primer 5′-TGGCAAACTGTGATGGACGA-3′ and reverse primer 5′- GGTTTCCACTATCGGCGAGT-3′. The membrane was hybridized overnight with the DIG-labeled *hpt* probe, washed with sequential low (2X SSC, 0.1% SDS) and high (0.5 X SSC, 0.1% SDS) stringency washes, and blocked with 1X blocking buffer (Roche, Branford, United States). The membrane was treated with anti-DIG AP Fab Fragments (Roche, Branford, United States) prepared in blocking buffer and washed three times with 1X washing buffer (1X maleic acid buffer, Tween 20). Detection was performed using the Tropix CDP-Star reagent (Applied Biosystems, Foster City, United States) for 5 min, undeveloped film exposed overnight and developed for imaging.

### Identification of Single Copy T-DNA Integration Events in T_2_–T_3_ Generations

Both single and multiple T-DNA copy insertion lines identified by Southern blot hybridization were tracked through sexual generations using Mendelian segregation ratios to produce homozygous lines. Mendelian segregation ratios of T_1_ families were determined by PCR screening for presence of *hpt* and tested for goodness of fit for a 3:1 segregation by chi squared analysis. Progeny from transgenic lines possessing single T-DNA integrations were PCR tested for *hpt* marker segregation in the subsequent T_2_ generation to identify homozygous lines. Single copy homozygous families were advanced, genotyped, and phenotyped to the T_3_ generation.

### Phenotypic Characterization of Transgenic Events

T_1_ families from three independent transgenic events plus EVC were selfed to obtain single T-DNA copy homozygous families, propagated, and subjected to phenotypic assessment up to the T_3_ generation. In each generation the *S. viridis* families were grown for 7 weeks and subjected to no watering for 1 week prior to harvesting. Plant growth parameters including total grain weight, grain number, panicle number, and primary panicle length were documented as a measure of agronomic performance at maturity. Plant development parameters including onset of flag leaf, inflorescence initiation, anthesis, seed setting, and senescence were documented in the T_3_ generation. For shoot dry matter content (DMC) determination, above ground parts of mature plants were harvested immediately after seed collection, placed in paper bags, and oven dried at 30°C for 1 week before assessing for DMC.

### Data Analysis

Statistical differences in agronomic traits between the transgenic T_3_ families and controls were analyzed by a student *t*- test and the data presented as mean ± standard error of mean (SEM), with ^∗^*p* ≤ 0.05, ^∗∗^*p* ≤ 0.01 and ^∗∗∗^*p* ≤ 0.001.

## Results

### Identification of *Atcel1* (AT1G70710.1) Homologs in *Setaria italica*

**FIGURE 1:**
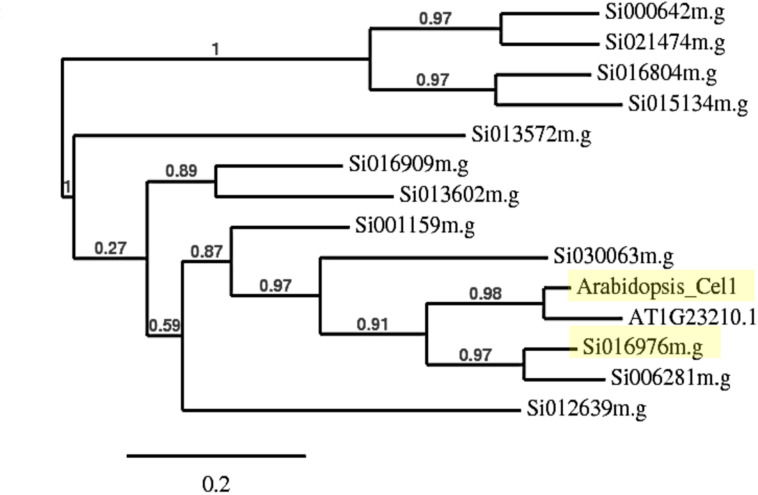
Neighbor-joining phylogenetic tree of *Atcel1* homologs in *S. italica*. The relative branch lengths indicate that Si016976m.g is more closely related to *Atcel1* (both highlighted in yellow) and therefore referred to as *Sicel1*.

Phylogenetic analysis of endo-1, 4-β-glucanase gene families in *S. italica* using AtCEL1 amino acid sequence as a query, revealed two closely related genes, *Si016976m.g* and *Si006281m.g*, with pairwise scores of 71.34 and 70.93%, respectively (Figure 1). BLASTP analysis indicated that Si016976m.g (Score: 703.7) was more closely related to *Atcel1* than Si006281m.g (Score: 688.3). Additional analysis using phylogeny.fr illustrated a parallel relationship between *Atcel1* and its closest homolog (At1G23210.1), compared to Si016976m.g and Si006281m.g. Si016976m.g is less evolutionarily divergent than Si006281m.g, and more similar to *Atcel1*. Thus, Si016976m.g was henceforth referred to as *Sicel1*.

### Generation, Selection and Molecular Analysis of Transgenic *S. viridis* Events

**FIGURE 2:**
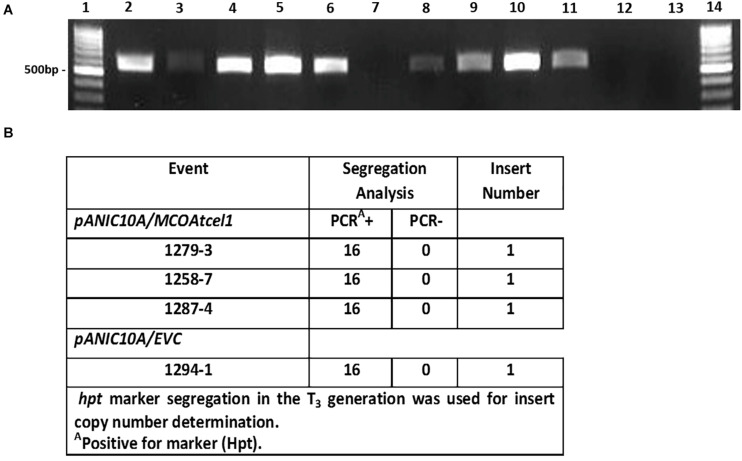
Molecular characterization of transgenic *MCOAtcel1* events **(A)**. Confirmation of *MCOAtcel1* transgene expression in 10 independent transgenic *S. viridis MCOAtcel1* T_0_ events (lane 2–11). The empty vector control T_0_ events tested negative for MCO*Atcel1* transcript (lane. 12–13). Lane 1 and 14 1 kb marker DNA. **(B)** PCR-confirmation of single insert status of transgenic T_3_ families using hygromycin phosphotransferase (*hpt*) marker gene segregation.

Sequence-confirmed *pANIC10A/MCOAtcel1* and *pANIC10A/Sicel1* constructs plus the *hpt* selectable marker-only control (EVC) were used to generate transgenic plants of *S. viridis*, accession A10.1 (Van Eck and Swartwood, 2015). Ten, seven, and two independent transgenic events were recovered for *pANIC10A/MCOAtcel1*, *pANIC10A/Sicel1*, and EVC constructs, respectively. RT-PCR was performed, either with *MCOAtcel1* specific primers (Figure 2A) or *hpt* (Supplementary Figure S1) primers, to confirm transgenic status of the plantlets. PCR-confirmed transgenic events were grown to maturity in soil and selfed. At each generation the *S. viridis* families were grown for 7 weeks, after which plant growth and development parameters were documented. None of the seven-pANIC10A/*Sicel1* transgenic events recovered displayed discernable phenotypic differences in comparison to EVCs and wild type A10.1 controls at the T_1_ and T_2_ generations, and therefore were not studied further. In contrast, progeny from the three independent high *MCOAtcel1* mRNA expressing events, 1287.4, 1258.7, and 1279.3 were characterized by enhanced height and vigor at both T_1_ and T_2_ generation (results not shown). Southern blot hybridization of T_1_ generation plants (Supplementary Figure S2) and PCR-based *hpt* marker segregation in the subsequent T_3_ generation (Figure 2B) were used to identify homozygous, single-copy transgenic lines in the T_3_ generation for further detailed trait analysis.

### Agronomic Trait Characterization of Transgenic *MCOAtcel1* T_3_ Events

**FIGURE 3:**
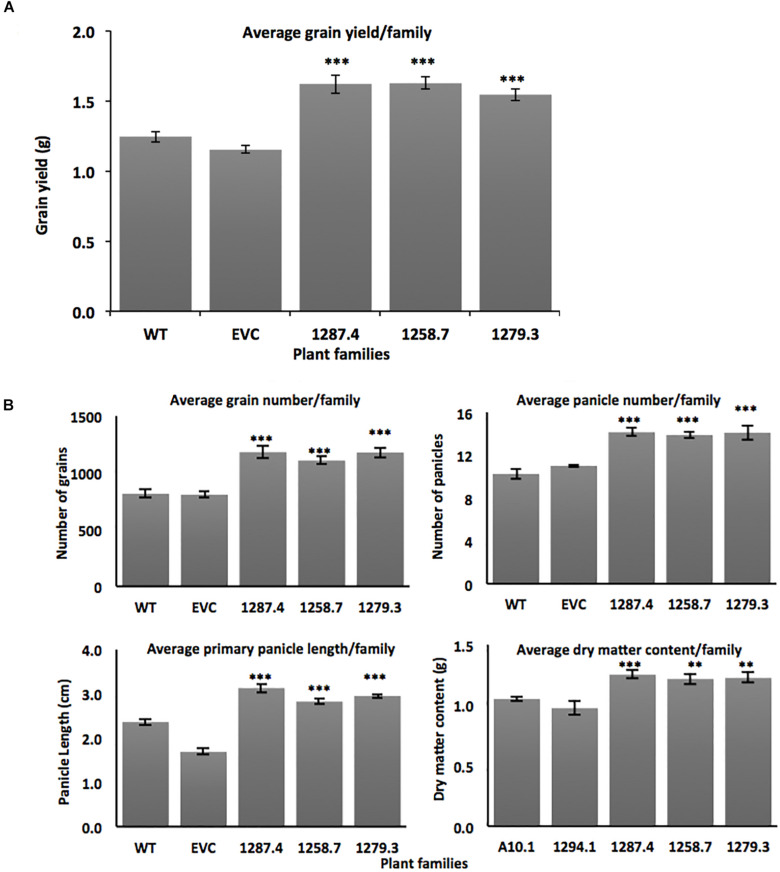
Agronomic trait characterization of transgenic *MCOAtcel1* T_3_ events **(A)** Elevated grain yield in T_3_ *S. viridis* families transgenic for *MCOAtcel1* in comparison to controls (*n* = 16 per family). **(B)** Elevated grain number, panicle number, primary panicle length, and vegetative dry matter content in T_3_ *S. viridis* families *MCOAtcel1* in comparison to controls (*n* = 16 per family). Data are shown as mean ± SEM, with *p* ≤ 0.001(***)/*p* ≤ 0.01(**)/*p* ≤ 0.05(*). EVC (empty vector control) and WT (wild type A10.1).

Sixteen individuals from the single-copy homozygous T_3_ *MCOAtcel* mRNA-expressing families from the three independent events 1287.4, 1258.7, and 1279.3 were grown to maturity and compared to the wild type A10.1 and EVC controls for primary panicle length, panicle number, grain number, total grain weight, and shoot dry matter content. Increased vigor and grain yield was observed in T_3_ plants from all three transgenic events. Grain yields increased significantly (*p* ≤ 0.001) by up to 26%, from an average of 1.19 ± 0.034 grams per plant in the controls to 1.62 ± 0.066 (26% gain), 1.63 ± 0.045 (26% gain), and 1.54 ± 0.045 (22% gain) per plant in the respective T_3_ families (Figure 3A). No change was seen in grain size, but assessment of grain yield components determined that total grain number increased (*p* ≤ 0.001) from an average of 818 ± 32 seeds per plant in controls to 1182 ± 52 (30% gain), 1108 ± 30 (26% gain), and 1179 ± 43 (30% gain) seeds per plant in the respective T_3_ families (Figure 3B).

We observed that yield increase was driven by an elevated number of seed-bearing panicles in the high *MCOAtcel1* expressing T_3_ families. Seed-bearing panicle number increased significantly (*p* ≤ 0.001) from an average of 10 ± 0.32 per plant in the controls to 14 ± 0.34 (30% gain), 13 ± 0.31 (24% gain), and 14 ± 0.77 (30% gain) for panicles in the respective T_3_ families compared to controls (Figure 3B). Similarly, length of the primary panicle was significantly elevated (*p* ≤ 0.01) in the high *MCOAtcel1* expresser T_3_ families, increasing from 2.1 cm ± 0.07 in controls to 3.1 ± 0.10 (32% gain), 2.8 ± 0.05 (25% gain), and 2.9 ± 0.04 (28% gain) cm per plant in the respective T_3_ families (Figure 3B). In addition to reproductive tissues, total vegetative shoot DMC, assessed after 7 days oven drying, was found to have increased significantly (*p* ≤ 0.01) in *Atcel1* expressing plants. While controls averaged 1.01 g ± 0.037 per plant, the transgenic 1287.4, 1258.7, and 1279.3 families averaged 1.25 g ± 0.033 (19% gain), 1.21 g ± 0.042 (16% gain), and 1.22 g ± 0.041 (17% gain) per plant, respectively (Figure 3B).

**FIGURE 4:**
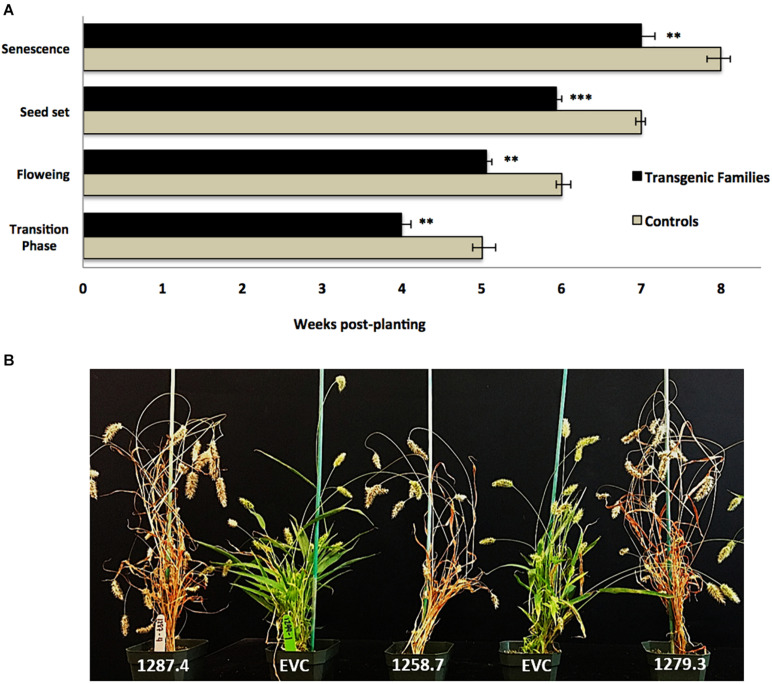
Developmental characterization of transgenic *MCOAtcel1* T_3_ events **(A)** Accelerated developmental transitions in T_3_ *S. viridis* families transgenic for *MCOAtcel1*. Data are an average of all three transgenic families in comparison to controls. Data are shown as mean ± SEM, with *p* ≤ 0.001(***)/*p* ≤ 0.01(**)/*p* ≤ 0.05(*). **(B)** Early maturation of T_3_ *S. viridis* families transgenic for *MCOAtcel1* in comparison to controls 7 weeks post-transplantation. On average plants of transgenic families completed their life cycle and reached senescence 7 days ahead of the empty vector (EVC), and wild-type A10.1 controls.

Plants from T_3_ families were also characterized by a statistically significant pattern of accelerated developmental transitions. *MCOAtcel1*-expressing T_3_ families transitioned from the vegetative to reproductive phase earlier than control families, as characterized by early onset of flag leaf (transition phase), inflorescence initiation and anthesis (flowering), seed setting, and senescence. On average, the T_3_ families completed these transition events 7 days earlier than the controls (Figure 4A), representing a 12.5% reduction in life span of 56 to 49 days from germination to maturity (Figure 4B).

## Discussion

*Atcel1* belongs to the GH9B sub-family and is thought to be involved in cell wall loosening that drives anisotropic cell enlargement, a central process regulating the kinetics of plant growth and development (Buchanan et al., 2012; Lipchinsky, 2013). Endo-1,4-β-glucanases (glycosyl hydrolases) facilitate cell wall loosening by breaking down 1,4-β-glucosyl linkages and cell wall polysaccharides (Buchanan et al., 2012). The decreased cross-linking is proposed to increase cell wall plasticity which in turn leads to accelerated plant growth (Park et al., 2003). Phylogenetic analysis revealed two closely related genes, *Si016976m.g* and *Si006281m.g* in *S. italica* (Figure 1). Further analysis indicated Si016976m.g to be the closest homolog to *Atcel* and was designated as *Sicel1*.

Transgenic overexpression of *Sicel1* did not confer a distinct phenotype in *S. viridis.* This was consistent with previous observation for overexpression of *Atcel1* and *PaPopCel1* in Arabidopsis and poplar, respectively (Park et al., 2003; Shani et al., 2004), and could be due to the homologous transgene-silencing (Matzke and Matzke, 1995). Conversely, transgenic *S. viridis* plants expressing the *MCOAtcel1* under control of the robust, constitutive ZmUBI promoter displayed enhanced biomass, grain yield, and early maturity. *MCOAtcel1* expressing plants were characterized by a 16–19% shoot biomass increment (Figure 3B), in a manner similar to the 20% enhancement in biomass production due to overexpression of *Atcel1* in poplar (Shani et al., 2004). Enhanced biomass is a good indicator of high vigor and grain yield in cereal grasses (Capstaff and Miller, 2018). Indeed, plants from T_3_ families from all three independent *MCOAtcel1* events studied displayed significantly increased grain weight, grain number, and panicle number (Figures 3A,B) compared to controls. Grain yield in cereals depends on component traits such as panicle length, panicle number, and grain weight (Reddy et al., 2013). Consistent with the above, we observed a 25–32%, 24–30%, and 26–30% increment in the component traits of primary panicle length, the total number of panicles, and total grain number, respectively, in the three *MCOAtcel1* events (Figure 3B). The improvement of yield components resulted in up to a 26% increase in the grain yield (Figure 3A), confirming for the first time that increased seed yields can be achieved by heterologous up regulation of *cel1*.

Accelerated maturity due to overexpression of *Atcel1* resulting in a 25% reduction in the time to harvest was reported in poplar and eucalyptus (Ledford, 2014). A similar response was observed in *S. viridis*. T_3_ families displayed accelerated growth and developmental trajectories, resulting in a 12.5% reduction in life span from germination to maturity (Figure 4A). Accelerated growth was statistically significant and likely due to early onset of juvenile-adult vegetative, adult vegetative-reproductive, and reproductive senescence transitions in the T_3_ families, in comparison to controls (Figures 4A,B).

Plant growth is driven by both cell division and cell expansion (Sun et al., 2017). Heterologous overexpression of Endo-1, 4-β- glucanases have previously been reported to result in expansion growth by trimming the disordered 1, 4-β- glucans (Park et al., 2003). Such expansion growth possibly resulted in the enhanced shoot biomass and accelerated growth observed in all three *MCOAtcel1* overexpression events characterized in our study (Figures 3B, 4A). The enhanced shoot biomass in part could also be due to an increase in the leaf number and size that presumably results in higher grain yield due to increased capacity for photosynthesis, as reported previously (Weraduwage et al., 2015; Driever et al., 2017).

Accelerated maturity provides opportunities for reduced exposure to post-anthesis stress factors (Vadez et al., 2012) and enables development of crops for climate-smart agriculture. For example, early-maturing and high-yielding primary cereal crops could provide farmers with greater flexibility under increasingly uncertain climatic conditions to sustainably increase productivity and contribute to national food security. In developing country agricultural systems, such flexibility also helps mitigate poverty by enabling subsistence farmers to access off-farm revenue-generating employment (Kidane et al., 2006).

Heterologous overexpression of *MCOAtcel1* caused enhanced grain yield, shoot biomass, and maturation rate in the model grass species *S. viridis* under growth chamber conditions. The agronomic trait differences observed were consistent with previous observations for dicots (Shani et al., 2004; Ledford, 2014), but are the first described in a monocot species. Importantly, increased seed yields are also achieved by overexpression of *cel1*. Our findings suggest that manipulation of *Atcel1* has the potential for developing early-maturing, higher-yielding, monocot cereals and biomass crops that could be suitable for climate-smart agriculture. Studies should now be performed to evaluate if similar yield enhancements and accelerated maturation can be achieved in economically important cereal species under field conditions.

## Data Availability Statement

The datasets generated for this study are available on request to the corresponding author.

## Author Contributions

BV directed the research, designed and conducted experiments, data analysis, and presentation, and wrote the manuscript. RP conducted experiments, collected data, and wrote manuscript. RW and AF conducted experiments. DB and SR conducted experiments and collected data. NT participated in data analysis and presentation and wrote the manuscript. All authors contributed to the article and approved the submitted version.

## Conflict of Interest

The authors declare that the research was conducted in the absence of any commercial or financial relationships that could be construed as a potential conflict of interest.

## Footnotes

1 www.phytozome.net

## Supplementary Material

Supplementary Figure 1 Molecular characterization of T_0_ *Sicel1* and EVC events showing transgenic nature of the regenerated plants. PCR confirmation of hygromycin phosphotransferase (*hpt*) marker gene in 7 independent transgenic *S. viridis Sicel1* (lane 3–9), and 2 empty vector controls (in duplicate, lane 13–16) T_0_ events. The *pANIC10A* vector and no template were used as positive (Lane 2, 10 and 11) and negative (Lane 12) controls, respectively, for the PCR. Lane 1, 1 kb marker DNA.

Supplementary Figure 2 Confirmation of transgene integration. Southern blot analysis confirming T-DNA integration and copy number of the *hpt* marker gene in *S. viridis* plants of 1258.7, 1279.3, and 1287.4 T_1_ families and empty vector controls. The genomic DNA was digested with *Mfe*1 enzyme and probed with DIG labeled *hpt* probe.
