# Supplementary figures and images for: Heterologous Overexpression of Arabidopsis cel1 Enhances Grain Yield, Biomass and Early Maturity in Setaria viridis
Source: Front Plant Sci. 2020 Nov 10;11:515078. doi: 10.3389/fpls.2020.515078 (PMC7683425; doi:10.3389/fpls.2020.515078)

## Slide 1
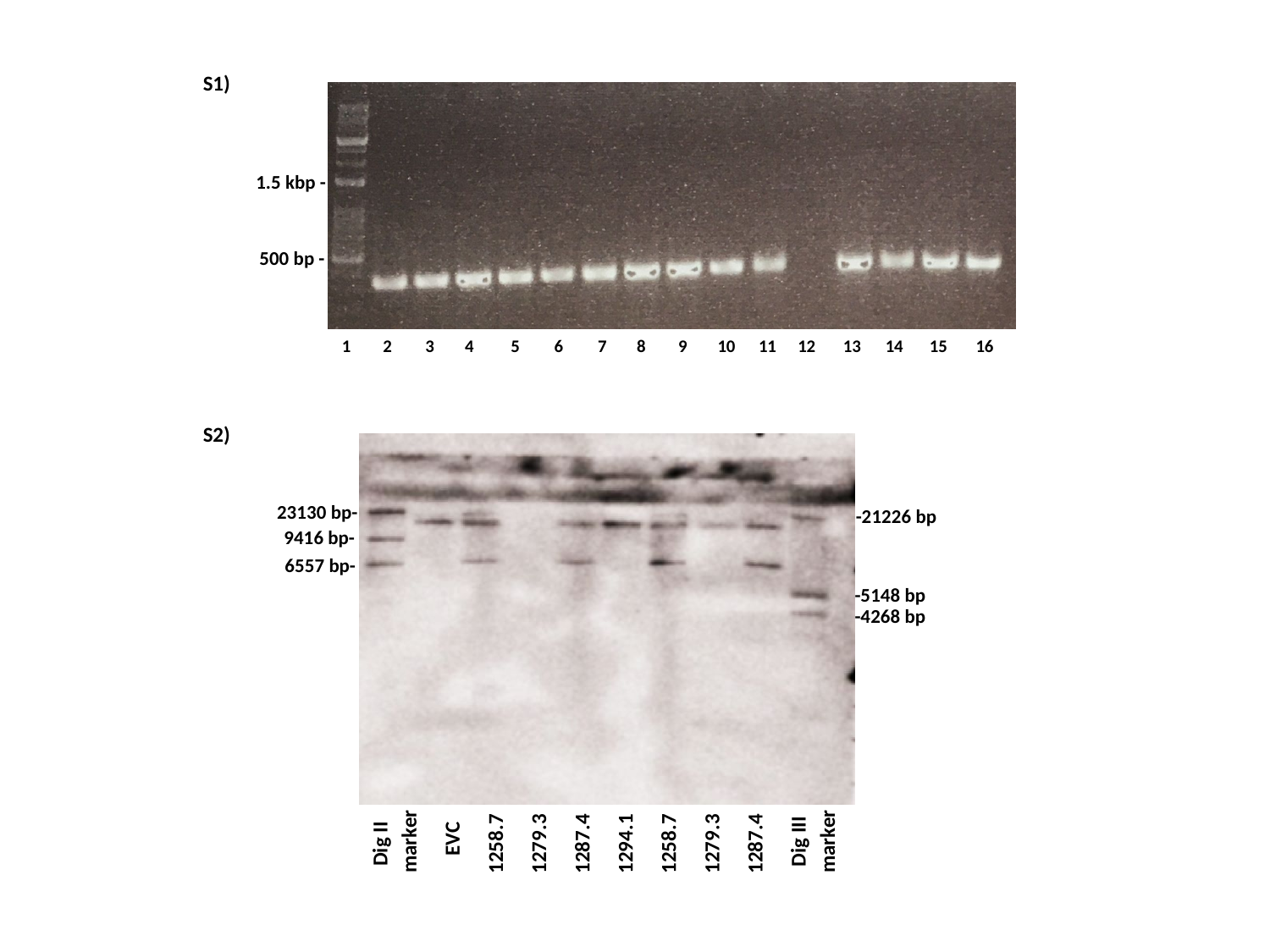

S1)
15
9
2
1
16
8
11
12
4
10
3
6
7
13
14
5
1.5 kbp -
500 bp -
S2)
23130 bp-
9416 bp-
6557 bp-
-21226 bp
-5148 bp
-4268 bp
Dig II marker
 EVC
1258.7
1279.3
1287.4
1294.1
1258.7
1279.3
1287.4
Dig III marker

Supplement: Supplementary Figure 1 — Molecular characterization of T0 Sicel1 and EVC events showing transgenic nature of the regenerated plants. PCR confirmation of hygromycin phosphotransferase (hpt) marker gene in 7 independent transgenic S. viridis Sicel1 (lane 3–9), and 2 empty vector controls (in duplicate, lane 13–16) T0 events. The pANIC10A vector and no template were used as positive (Lane 2, 10 and 11) and negative (Lane 12) controls, respectively, for the PCR. Lane 1, 1 kb marker DNA. [file Presentation_1.PPTX]
